# Comparative Progression of Precancerous Gastric Lesions in Autoimmune and *Helicobacter pylori*-Associated Gastritis: A Longitudinal Cohort from Eastern Europe

**DOI:** 10.3390/medsci14060661

**Published:** 2026-10-11

**Authors:** Ana-Maria Filip, Sabrina-Nicoleta Munteanu, Simona Mocan, Patrick-Lazăr-Dominik Chiciudean, Luciana Arjoca, Cristina Monica Pantea, Marian-Ioan Filip, Anca Negovan

**Affiliations:** 1 Department of Internal Medicine I, Emergency County Hospital of Targu Mures, 540136 Targu Mures, Romania; ana-maria.filip@umfst.ro (A.-M.F.); anca.negovan@umfst.ro (A.N.); 2 Department of Clinical Science—Internal Medicine, “George Emil Palade” University of Medicine, Pharmacy, Science, and Technology of Targu Mures, 540139 Targu Mures, Romania; 3 Pathology Department, Emergency County Hospital of Targu Mures, 540136 Targu Mures, Romania; 4 Oncology Department, County Clinical Hospital of Targu Mures, 540140 Targu Mures, Romania; 5 Faculty of Medicine, “George Emil Palade” University of Medicine, Pharmacy, Science, and Technology of Targu Mures, 540139 Targu Mures, Romania; 6 Gastroenterology Department, Emergency County Hospital of Targu Mures, 540136 Targu Mures, Romania; 7 Grigore T. Popa University of Medicine and Pharmacy Iasi, 700115 Iasi, Romania

**Keywords:** autoimmune gastritis, *Helicobacter pylori*, intestinal metaplasia, disease progression, precancerous conditions

## Abstract

Background: Autoimmune gastritis (AIG) and *Helicobacter pylori* gastritis are distinct etiologies of chronic gastritis, yet comparative data on their long-term progression remain limited. Objectives: We compared the evolution of precancerous gastric lesions in patients with AIG and *H. pylori*-associated gastritis. Methods: This retrospective single-center cohort included 116 patients (52 histologically defined AIG, 64 *H. pylori*-associated gastritis) who underwent two upper endoscopies with Sydney-protocol biopsies at least 12 months apart between 2013 and 2024 (median follow-up 60 and 49 months). The primary outcome was the development of glandular atrophy or intestinal metaplasia (IM) not present at baseline. Results: New atrophy or IM developed in 61.5% (32/52) of AIG and 34.4% (22/64) of *H. pylori* patients (adjusted odds ratio [OR] 3.81, 95% confidence interval [CI] 1.53–9.49). Incident atrophy was more frequent in AIG (65.1% vs. 31.5%), including in comparison with baseline *H. pylori*-positive patients only (adjusted OR 3.02, 95% CI 1.08–8.46), whereas differences in IM were less consistent. Progression occurred in 42.9% of patients who were *H. pylori*-negative after treatment and 55.6% of persistently infected patients (*p* = 0.70). Proton pump inhibitor (PPI) use, an exploratory clinical risk score, and symptom changes were not associated with progression. Conclusions: In this selected cohort with repeat endoscopy, histologically defined AIG was associated with more frequent histological progression, particularly glandular atrophy, than *H. pylori*-associated gastritis. Prospective studies with serologically confirmed AIG and neoplastic endpoints are needed to inform risk stratification.

## 1. Introduction

Chronic gastritis can progress through well-defined precancerous stages toward gastric adenocarcinoma via two predominant pathways: *Helicobacter pylori* infection and autoimmune mechanisms [1,2]. While both share similar histological endpoints, including glandular atrophy and intestinal metaplasia, emerging evidence suggests fundamental differences in their natural history, progression rates, and reversibility potential [3,4]. Understanding these differences has gained importance as gastric cancer incidence patterns shift globally, with Eastern European countries demonstrating persistently elevated rates despite declining trends elsewhere [3,4]. Current guidelines emphasize *H. pylori* eradication as the cornerstone of prevention, with substantial evidence supporting histological regression after eradication, particularly before extensive lesions develop [5,6]. This predictable trajectory and demonstrated reversibility have enabled standardized surveillance protocols.

Autoimmune gastritis presents a fundamentally different entity characterized by immune-mediated parietal cell destruction, resulting in corpus-predominant atrophy, hypochlorhydria, and hypergastrinemia [7,8]. Despite affecting 18% of patients with gastric precancerous lesions, most cases remain unrecognized due to insidious presentation and lack of routine antibody screening [9]. Natural history data remain limited compared to decades of *H. pylori* research, leaving fundamental questions unanswered regarding progression rates, reversibility, and optimal surveillance strategies. Current guidelines explicitly acknowledge these gaps, noting that “the optimal surveillance interval for individuals with AIG is unclear” and recommending “individualized assessment” without evidence-based criteria for such individualization [2]. The MAPS III guideline (Management of Epithelial Precancerous Conditions and Early Neoplasia of the Stomach) recommends 3-year intervals for advanced atrophic gastritis while acknowledging that regional adaptation may be necessary [6]. These knowledge gaps carry particular urgency in Eastern Europe, where gastric cancer incidence remains 2–3-fold higher than in Western Europe [3]. Cancer outcomes differ markedly across Europe, with persistent disparities in mortality between countries [10].

We hypothesized that AIG would demonstrate higher progression rates to intestinal metaplasia and advanced atrophy compared to *H. pylori* gastritis, with reduced reversibility. We further hypothesized that proton pump inhibitors (PPIs) would differentially accelerate progression in AIG through hypergastrinemia-driven mechanisms, and that symptom severity would correlate with histological progression, enabling clinical risk stratification.

To test these hypotheses, we conducted a longitudinal comparative analysis in a high-risk Eastern European population. Our objectives were to: (1) quantify and compare progression rates between gastritis types; (2) assess lesion reversibility following *H. pylori* eradication versus standard autoimmune management; (3) evaluate differential PPI impact on progression; (4) analyze symptom–histology correlations as progression predictors; and (5) inform etiology-based risk stratification.

## 2. Materials and Methods

### 2.1. Study Design and Setting

This retrospective observational cohort study was conducted at a single internal medicine center in Târgu Mureș, Romania, between January 2013 and December 2024. Patients were identified from the endoscopy registry of our internal medicine clinic and comprised patients followed in our department. A total of 116 patients with autoimmune gastritis (AIG) or *H. pylori* gastritis who underwent two upper endoscopies with systematic gastric biopsies were followed for a median of 60 months (AIG) and 49 months (*H. pylori* gastritis) to evaluate the evolution of chronic gastritis and precancerous lesions.

### 2.2. Study Population

**Figure 1:**
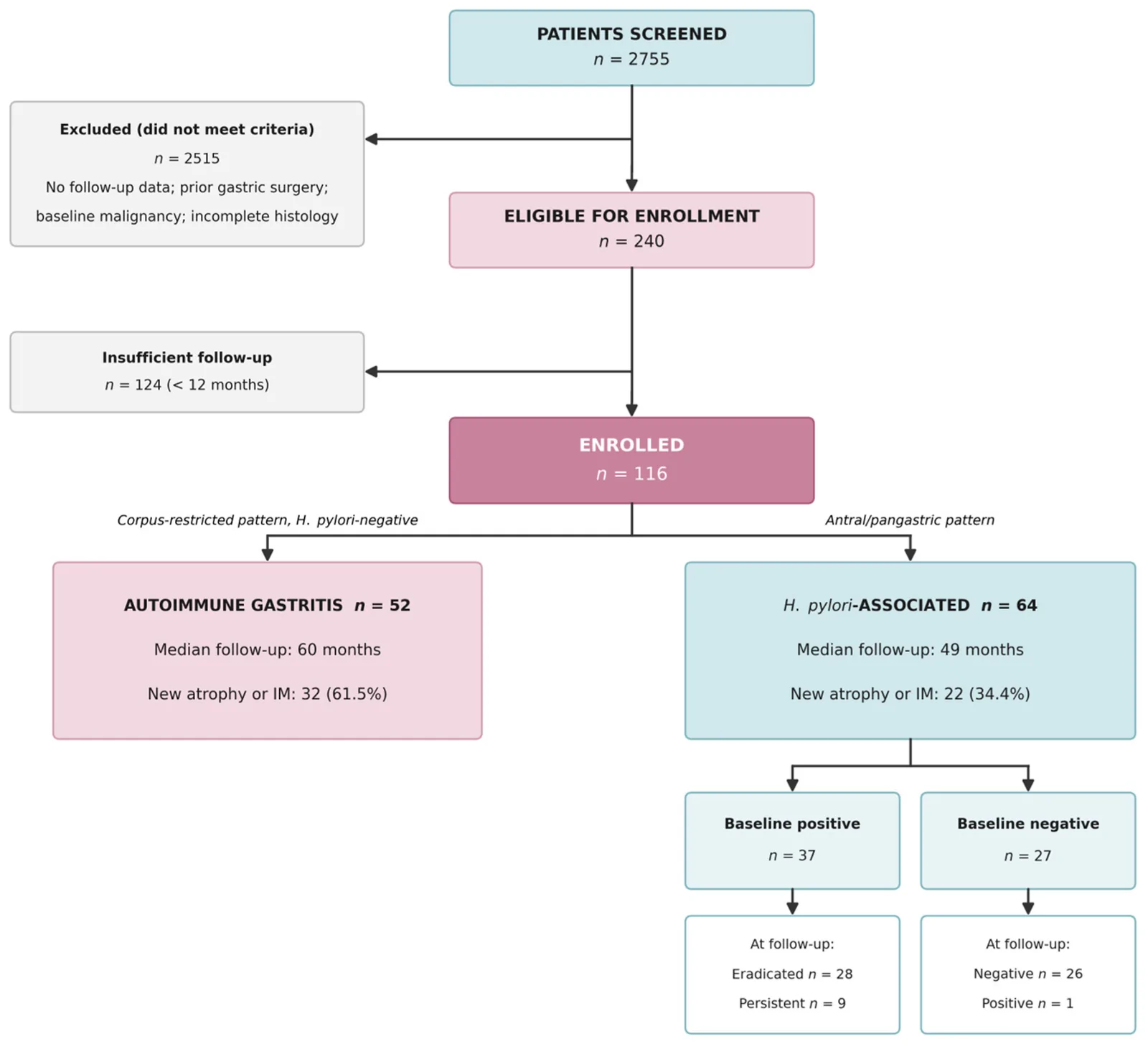
Patient selection and enrollment flowchart. Screening, eligibility assessment, and final cohort classification based on histological pattern and *H. pylori* status; baseline *H. pylori*-positive and -negative patients are shown by infection status at follow-up.

Inclusion criteria were age ≥ 18 years, baseline and follow-up upper endoscopy (≥12 months apart) with systematic biopsies, complete histopathological evaluation according to the updated Sydney System, and available clinical data. Exclusion criteria included previous gastric surgery or endoscopic resection, baseline gastric cancer, incomplete histology or clinical data, follow-up <12 months, and equivocal topographic gastritis patterns. For patients with more than two endoscopies, only the first two examinations performed at least 12 months apart were analyzed. The most frequent reason for exclusion among screened patients was the absence of a second endoscopy (Figure 1).

### 2.3. Endoscopic Protocol and Histological Assessment

Upper endoscopy was performed using high-definition white-light endoscopy (Olympus EVIS EXERA III and EVIS X1 systems, Olympus Corp., Tokyo, Japan); narrow-band imaging was used in part of the examinations. Five biopsies (two antral, two corpus, one incisura angularis) were obtained according to the updated Sydney System at every examination throughout the study period. Histological evaluation was performed by a single experienced gastrointestinal pathologist using hematoxylin-eosin staining, with Giemsa for *H. pylori* and PAS/Alcian blue for intestinal metaplasia when indicated. Chronic inflammation and neutrophilic activity were graded according to the updated Sydney System; glandular atrophy, intestinal metaplasia, and *H. pylori* infection were recorded as present or absent at each biopsy site, and lesion topography was recorded. Severity grades of atrophy and intestinal metaplasia were not recorded. Biopsies were assessed as part of routine clinical care; the pathologist was not blinded to the endoscopic findings or previous histology, and slides were not re-reviewed for this study.

### 2.4. Group Classification

Patients were classified according to histological distribution and *H. pylori* status.

The AIG group included patients negative for *H. pylori* at baseline with corpus-predominant or corpus-restricted inflammation, atrophy, or intestinal metaplasia and no antral-predominant or pangastric involvement; this group is referred to as histologically defined AIG. The *H. pylori*-associated group included patients with antral-predominant or pangastric involvement who were either *H. pylori*-positive on baseline histology (*n* = 37; confirmed by serology or stool antigen testing before endoscopy in some of them) or *H. pylori*-negative at baseline with an antral-predominant or pangastric pattern consistent with previous infection (*n* = 27). Cases with corpus-predominant gastritis despite *H. pylori* positivity were excluded to minimize misclassification. Group assignment was based exclusively on the topography of inflammatory and precancerous lesions at the baseline endoscopy and was completed before follow-up data were entered into the database, so that classification was blinded to follow-up findings. All AIG patients remained *H. pylori*-negative at follow-up.

All *H. pylori*-positive patients received 14-day standard triple therapy (PPI, amoxicillin, clarithromycin). Eradication was assessed histologically at the follow-up endoscopy rather than by a timed test of cure, because recruitment began before universal test-of-cure recommendations.

### 2.5. Clinical Data Collection

Clinical data were extracted independently by two investigators from electronic medical records. Variables included demographics, smoking status, symptoms, PPI use (continuous use for ≥6 months at the time of the index endoscopy versus intermittent/no use), comorbidities, and laboratory parameters including hemoglobin, mean corpuscular volume, vitamin B12, and folate when available. Symptoms were recorded as present or absent and summed into a dyspeptic score (epigastric pain, heartburn, early satiety, nausea; range 0–4) and a systemic score (fatigue, weight loss; range 0–2). An exploratory composite risk score (range 0–7; one point each for age > 50 years, continuous PPI use, current smoking, diabetes, autoimmune thyroiditis, anemia, and nonsteroidal anti-inflammatory drug (NSAID) use) was constructed by the authors. Neither score is a validated instrument; both were used for exploratory purposes only. Endoscopic and histological findings and symptoms were recorded separately at both endoscopies; all other clinical variables, including PPI use, refer to the index endoscopy.

### 2.6. Outcome Variables

The primary outcome was histological progression, defined as the development at follow-up of glandular atrophy or intestinal metaplasia not present at baseline. Because severity grades were not recorded, worsening of pre-existing lesions and Operative Link on Gastritis Assessment/Operative Link on Gastric Intestinal Metaplasia (OLGA/OLGIM) stages could not be assessed. Secondary outcomes included incident atrophy (among patients without atrophy at baseline), incident intestinal metaplasia (among patients without intestinal metaplasia at baseline), lesions no longer detected at follow-up, symptom change, and *H. pylori* status at follow-up.

### 2.7. Statistical Analysis

Continuous variables were expressed as mean ± standard deviation or median (interquartile range [IQR]), according to Shapiro–Wilk normality testing. Group comparisons used Student’s *t*-test or Mann–Whitney U test for continuous variables and the chi-square test without continuity correction, or Fisher’s exact test when any expected count was <5, for categorical variables. Differences in proportions are reported as risk differences with Newcombe 95% confidence intervals (CI), and proportions with Wilson 95% CI. Because progression status was ascertained at a single follow-up endoscopy, the time of onset is unknown; the main adjusted analysis was therefore logistic regression, with results expressed as odds ratios (OR) and 95% CI, adjusted for age, sex, follow-up duration, baseline atrophy or intestinal metaplasia, PPI use, and smoking. Covariates were selected a priori for clinical relevance; follow-up duration was included to account for the different intervals between endoscopies. With 54 events and seven covariates (approximately eight events per variable), adjusted estimates are imprecise; a reduced model adjusted only for age, sex, and follow-up duration was therefore fitted as a sensitivity analysis. Kaplan–Meier curves (time = interval between endoscopies) with log-rank tests and Cox proportional hazards models (without follow-up duration as a covariate) are presented descriptively. Effect modification by PPI use was tested with a group-by-PPI interaction term. Sensitivity analyses restricted the comparator group to patients *H. pylori*-positive at baseline and restricted the cohort to PPI non-users. There were no missing values in the analyzed variables, because complete histological and clinical data were an inclusion criterion; laboratory parameters recorded inconsistently in routine care (vitamin B12, folate) were not analyzed, and analyses were complete-case. Within-group comparisons used paired *t*-tests or Wilcoxon signed-rank tests, while McNemar’s test analyzed paired categorical variables. Statistical significance was set at *p* < 0.05. Analyses were performed in R (version 4.3.0; R Foundation for Statistical Computing, Vienna, Austria) and Python (version 3.11; Python Software Foundation, Wilmington, DE, USA), using the statsmodels package.

### 2.8. Ethical Considerations

The study was approved by the Ethics Committee of the Emergency County Hospital of Targu Mures, Romania (No. Ad. 4194/29.05.2026) and was conducted in accordance with the Declaration of Helsinki, Good Clinical Practice, and STROBE guidelines. Patient anonymity was ensured by de-identifying all data before analysis, and written informed consent was obtained from all participants.

## 3. Results

### 3.1. Study Population and Baseline Characteristics

Between January 2013 and December 2024, 116 patients were included: 52 with AIG and 64 with *H. pylori*-associated gastritis (Figure 1). The *H. pylori* group included 37 patients (57.8%) with active infection at baseline and 27 patients (42.2%) who were *H. pylori*-negative but showed antral-predominant or pangastric patterns consistent with previous infection. Each patient contributed two endoscopies. Median follow-up was 60.0 months (IQR 21.0–74.0) for autoimmune and 49.0 months (IQR 34.8–72.0) for *H. pylori* gastritis (*p* = 0.874).

Baseline characteristics are presented in Table 1.

### 3.2. Progression Patterns and Comparative Analysis

**Figure 2:**
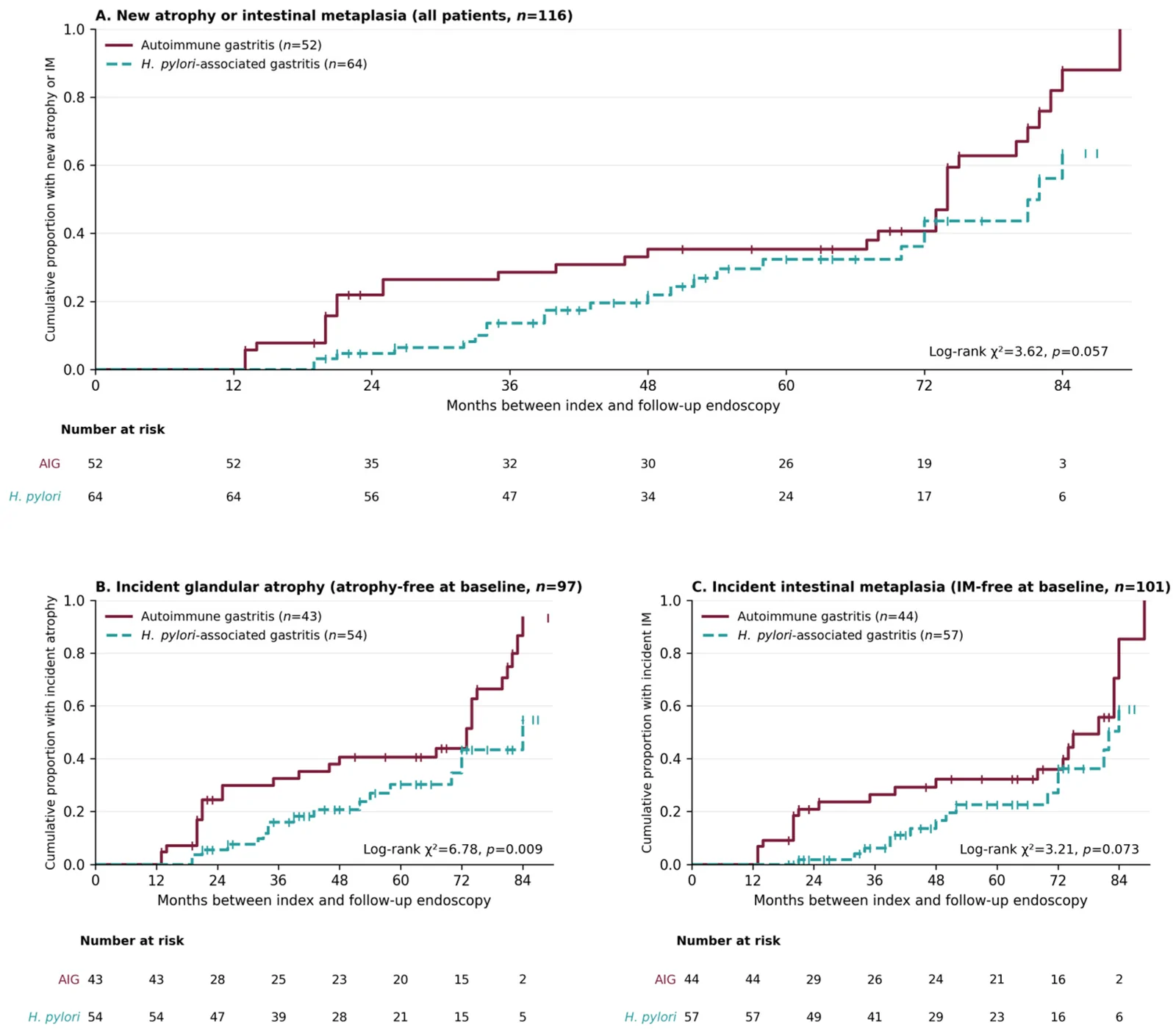
Cumulative proportion of patients with new lesions by gastritis type (Kaplan–Meier estimates, descriptive). (**A**) New atrophy or intestinal metaplasia, all patients (AIG *n* = 52, median follow-up 60 months, IQR 21–74; *H. pylori n* = 64, median follow-up 49 months, IQR 35–72); log-rank χ^2^ = 3.62, *p* = 0.057. (**B**) Incident glandular atrophy among patients without atrophy at baseline (*n* = 97: AIG 43, *H. pylori* 54); log-rank *p* = 0.009. (**C**) Incident intestinal metaplasia among patients without intestinal metaplasia at baseline (*n* = 101: AIG 44, *H. pylori* 57); log-rank *p* = 0.073. Time is the interval between the index and follow-up endoscopy; progression was ascertained only at the follow-up endoscopy. Tick marks indicate patients without the event; numbers at risk are shown below each panel.

The primary outcome, development of atrophy or intestinal metaplasia not present at baseline, occurred in 61.5% (32/52) of AIG patients and 34.4% (22/64) of *H. pylori* patients (risk difference 27.2 percentage points, 95% CI 8.9–43.1; χ^2^ = 8.51, *p* = 0.004; Table 2). The association persisted after adjustment for age, sex, follow-up duration, baseline atrophy or intestinal metaplasia, PPI use, and smoking (adjusted OR 3.81, 95% CI 1.53–9.49, *p* = 0.004; Table 3) and in the reduced model adjusted for age, sex, and follow-up duration (OR 3.06, 95% CI 1.41–6.63). The width of these intervals reflects the limited sample size. Among patients free of the respective lesion at baseline, incident atrophy occurred in 65.1% (28/43) vs. 31.5% (17/54) (adjusted OR 3.63, 95% CI 1.47–8.98) and incident intestinal metaplasia in 47.7% (21/44) vs. 26.3% (15/57) (adjusted OR 2.46, 95% CI 1.01–6.01). Cumulative incidence curves for each lesion are shown in Figure 2B,C (log-rank *p* = 0.009 for atrophy and *p* = 0.073 for intestinal metaplasia).

Kaplan–Meier curves are presented descriptively in Figure 2A. Because progression was ascertained only at the follow-up endoscopy, the interval between examinations is an upper bound of the time to progression, and the timing of repeat endoscopy was clinically determined; accordingly, progression proportions did not increase with longer intervals (AIG: 64.7% at <24 months vs. 57.7% at >60 months; *H. pylori*: 37.5% vs. 26.1%). The log-rank comparison did not reach statistical significance (χ^2^ = 3.62, *p* = 0.057).

In Cox regression without follow-up duration as a covariate, the hazard ratio for AIG versus *H. pylori*-associated gastritis was 1.71 (95% CI 0.98–2.97, *p* = 0.057) unadjusted and 1.81 (95% CI 1.00–3.28, *p* = 0.048) after adjustment for age, sex, baseline atrophy or intestinal metaplasia, PPI use, and smoking. The lower confidence limits lie at or below 1, so these time-to-event estimates are imprecise and only marginally support a faster rate of progression; the difference between groups is established for the proportion of patients with progression at follow-up rather than for time to progression. In sensitivity analyses (Table 2), restricting the comparator group to patients *H. pylori*-positive at baseline attenuated the difference in overall progression (61.5% vs. 45.9%; adjusted OR 2.36, 95% CI 0.83–6.66, *p* = 0.106) and in incident intestinal metaplasia (adjusted OR 1.75, 95% CI 0.64–4.75), whereas the excess of incident atrophy in AIG persisted (65.1% vs. 36.4%; adjusted OR 3.02, 95% CI 1.08–8.46, *p* = 0.036). The direction of the association was unchanged among PPI non-users (57.7% vs. 37.5%; adjusted OR 1.82, 95% CI 0.64–5.16).

### 3.3. Development and Regression of Precancerous Lesions

**Figure 3:**
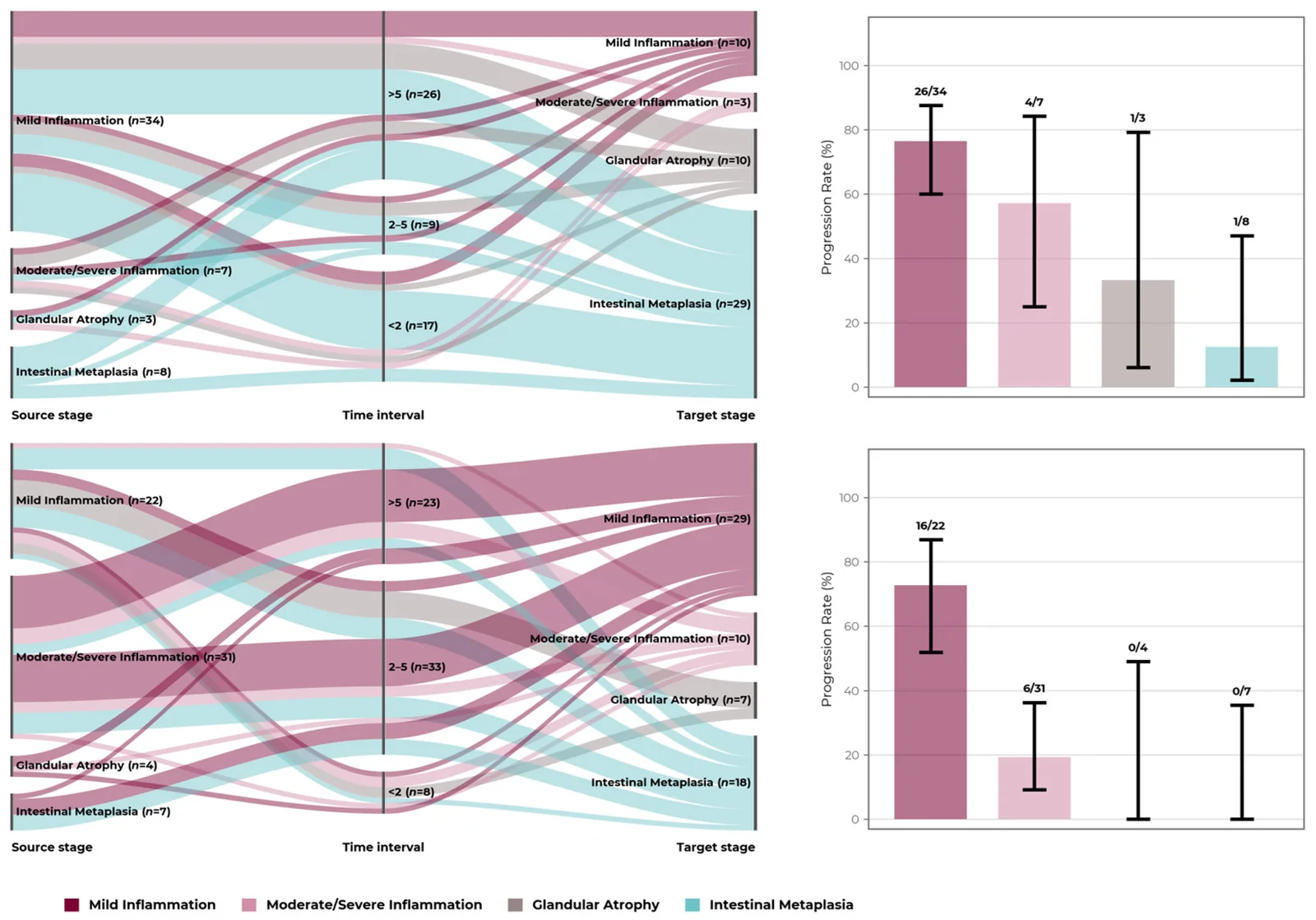
Histological progression patterns in autoimmune versus *H. pylori* gastritis. (**Left panels**): Sankey diagrams illustrate patient transitions from baseline stage (left) through follow-up intervals (middle: <2, 2–5, >5 years) to target stage (right), with flow width proportional to patient numbers, flow color indicating the target stage, and *n* shown for each stage and interval. Stages were assigned hierarchically from the recorded histology: intestinal metaplasia > glandular atrophy > moderate/severe inflammation (grade 2–3) > mild inflammation (grade 0–1). (**Right panels**): proportion of patients developing new atrophy or intestinal metaplasia by baseline stage (*n*/*N*), with 95% Wilson confidence intervals. AIG (top): *n* = 52, median follow-up 60 months (IQR 21–74); *H. pylori* gastritis (bottom): *n* = 64, median follow-up 49 months (IQR 35–72).

Table 4 presents detailed transitions in precancerous lesions from baseline to follow-up, with 95% CI for new lesion development; individual transitions between histological stages, with the number of patients in each stage and confidence intervals, are shown in Figure 3. Both gastritis types showed new lesions over follow-up, but AIG showed higher rates of new lesion development and lesions were rarely no longer detected at follow-up, resulting in greater net advancement.

### 3.4. H. pylori Eradication and Disease Evolution

Among the 37 patients with active *H. pylori* infection at baseline, all of whom received eradication therapy, 28 (75.7%) were *H. pylori*-negative at the follow-up endoscopy and 9 (24.3%) remained infected. Eradication was assessed histologically at the follow-up endoscopy. Of the 27 baseline-negative patients, one was *H. pylori*-positive at follow-up.

New atrophy or intestinal metaplasia developed in 42.9% (12/28) of patients who were *H. pylori*-negative after treatment, 55.6% (5/9) of persistently infected patients, and 15.4% (4/26) of baseline-negative patients who remained negative; the single newly infected patient progressed (Table 5). The difference between eradicated and persistently infected patients was not statistically significant (*p* = 0.70, Fisher’s exact test), and the confidence intervals of the subgroups overlap widely. Baseline atrophy or intestinal metaplasia was present in 3/28, 1/9, and 7/26 patients, and median follow-up was 48, 52, and 49 months, respectively. Because post-treatment confirmation was not systematic, eradication status reflects *H. pylori* status observed at the follow-up endoscopy rather than a verified treatment outcome. Progression therefore differed by observed infection status, but the subgroups were small, treatment success was not randomized, and the time of clearance is unknown; these observations should be interpreted cautiously and do not show that eradication caused lower progression.

### 3.5. Proton Pump Inhibitor Effects

Continuous PPI use at the time of the index endoscopy was documented in 36.2% (42/116) of patients, with significantly higher prevalence in the AIG group (50.0% vs. 25.0%, *p* = 0.005).

Contrary to our hypothesis that PPIs would differentially accelerate progression in AIG, no differential effect was observed. In AIG, new atrophy or intestinal metaplasia developed in 57.7% (15/26) of non-users and 65.4% (17/26) of PPI users (OR 1.39, 95% CI 0.45–4.25). In *H. pylori*-associated gastritis, the corresponding proportions were 37.5% (18/48) and 25.0% (4/16) (OR 0.56, 95% CI 0.16–1.99).

In logistic regression, the group-by-PPI interaction was not significant (OR 2.49, 95% CI 0.46–13.61, *p* = 0.291), and PPI use was not associated with progression in the fully adjusted model (OR 0.86, 95% CI 0.34–2.17, *p* = 0.750; Table 3). Given the small subgroups, these analyses were underpowered to exclude a modest PPI effect.

Because PPI use differed between groups, the analysis was repeated in PPI non-users, with an unchanged direction of association between gastritis type and progression (Table 2).

### 3.6. Clinical Risk Factors and Predictive Modeling

Several clinical risk factors were evaluated for their association with disease progression. Age over 50 years was highly prevalent (80.2%, 93/116) with similar distribution between groups. Anemia was significantly more common in AIG (48.1% vs. 15.6%, *p* < 0.001), while current smoking was more prevalent in *H. pylori*-associated gastritis (32.8% vs. 15.4%, *p* = 0.031).

In univariable logistic regression, no clinical factor was significantly associated with progression apart from baseline atrophy or intestinal metaplasia, which was inversely associated because these patients could not newly develop a pre-existing lesion (Table 3). Smoking was not associated with progression overall (OR 0.51, 95% CI 0.21–1.23) or within either group (AIG: 62.5% of smokers vs. 61.4% of non-smokers; *H. pylori*: 23.8% vs. 39.5%) and was included in all multivariable models; because smokers were concentrated in the *H. pylori* group, this imbalance would not explain the excess progression in AIG.

An exploratory composite risk score (0–7 points) was constructed incorporating age > 50 years, PPI use, smoking, diabetes, thyroiditis, anemia, and NSAID use. Score distribution was: 33.6% (39/116) 0–1 points, 53.4% (62/116) 2–3 points, and 12.9% (15/116) ≥4 points.

Progression rates by risk category showed minimal variation: 43.6% (17/39), 46.8% (29/62), and 53.3% (8/15), respectively. At patient level, there was no significant correlation between the risk score and progression (Spearman ρ = 0.066, *p* = 0.482; OR per point 1.15, 95% CI 0.81–1.62).

Receiver operating characteristic (ROC) curve analysis showed that this score had no meaningful discriminative ability (area under the curve [AUC] = 0.537, 95% CI 0.435–0.639). At a cut-off of ≥4 points, sensitivity was 14.8% and specificity 88.7%.

### 3.7. Symptom–Histology Correlations

Baseline dyspeptic symptom scores (sum of epigastric pain, heartburn, early satiety, and nausea; range 0–4) were similar between groups: 0.62 ± 0.63 in AIG and 0.55 ± 0.78 in *H. pylori*-associated gastritis (*p* = 0.298). At follow-up, dyspeptic scores were higher in the *H. pylori* group (autoimmune: 0.83 ± 0.81; *H. pylori*: 1.33 ± 0.89; *p* = 0.002).

Systemic symptom scores (fatigue and weight loss; range 0–2) were identical at baseline (0.08 ± 0.27 in both groups, *p* = 0.99) and similar at follow-up (0.27 ± 0.49 vs. 0.22 ± 0.42, *p* = 0.66).

Within-group paired analysis showed worsening of systemic symptoms in both groups (AIG *p* = 0.008; *H. pylori p* = 0.020) and of dyspeptic symptoms in the *H. pylori* group (*p* < 0.001), but not in AIG (*p* = 0.183).

Spearman correlation analysis was performed to assess whether symptom changes predicted histological progression. In AIG, correlations were weak and non-significant for both dyspeptic symptoms (ρ = −0.047, *p* = 0.742) and systemic symptoms (ρ = −0.037, *p* = 0.794). Similarly, in *H. pylori*-associated gastritis, correlations were minimal for dyspeptic symptoms (ρ = −0.119, *p* = 0.349) and systemic symptoms (ρ = 0.056, *p* = 0.663).

## 4. Discussion

### 4.1. Main Findings

Our findings show that histologically defined AIG was associated with more frequent development of precancerous lesions than *H. pylori*-associated gastritis (61.5% vs. 34.4%; adjusted OR 3.81), with minimal reversibility (atrophy no longer detected in 5.8% vs. 10.9%) and substantially greater net advancement of precancerous lesions (+48.1 vs. +15.6 percentage points for atrophy). The difference was most robust for glandular atrophy, which remained more frequent in AIG even when the comparator was restricted to patients with active infection at baseline, whereas differences in intestinal metaplasia were smaller and less certain. These etiology-specific differences align with the conceptual framework of the 2024 Real-world Gastritis Initiative (RE.GA.IN.) consensus, which reframed gastritis prognosis around topography and etiology rather than histology alone [11], and complement the OLGA/OLGIM staging logic underpinning current European recommendations [6,12]. This topography-driven distinction is corroborated by clinical outcome data showing that gastric cancers arising in AIG are preferentially localized to the proximal stomach—including the esophagogastric junction, fundus, and corpus—in contrast to the more antral-predominant pattern typical of *H. pylori*-associated malignancy [8].

### 4.2. Progression Rates in Regional Context

Our AIG progression rate (61.5%) refers to the appearance of atrophy or intestinal metaplasia and is therefore not directly comparable with the neoplastic event rates reported in Western European cohorts under surveillance, such as that described by Esposito et al. [13]. Nevertheless, the high frequency of new precancerous lesions in our cohort is relevant in Eastern Europe, where gastric cancer incidence remains elevated despite global declining trends [3,4].

In contrast, progression in our *H. pylori*-associated group (34.4%) was lower and heterogeneous, being most frequent in persistently infected and least frequent in baseline-negative patients, consistent with the eradication-focused paradigm underlying MAPS III recommendations [6].

### 4.3. H. pylori Eradication and Reversibility

Progression differed by observed infection status: it was most frequent with persistent infection (55.6%), intermediate in patients *H. pylori*-negative after treatment (42.9%), and least frequent in baseline-negative patients (15.4%), although the difference between eradicated and persistently infected patients was not statistically significant. Persistent infection may partly reflect the rising global prevalence of antibiotic-resistant *H. pylori* strains, which has increasingly undermined the reliability of standard eradication regimens [14]. Because eradication status was determined at the follow-up endoscopy rather than by a timed test of cure, the subgroups were small, and treatment success was not randomized, these data cannot show that eradication lowered progression risk; lesions present before eradication may also have contributed. In the Shandong intervention trial summarized in the RE.GA.IN. consensus, successful *H. pylori* treatment was associated with a higher likelihood of intestinal metaplasia regression than placebo (relative risk 1.55, 95% CI 1.03–2.33) [11]; in patients with advanced atrophic corpus gastritis, mean atrophy and intestinal metaplasia scores improved 2.5 years after eradication [15], and regression of intestinal metaplasia has been reported in approximately 20–44% of patients followed for 5–20 years after eradication [16]. Inflammatory infiltrate, however, resolves rapidly after eradication while atrophy and intestinal metaplasia frequently remain unchanged in the short term [17], which is consistent with the modest structural reversibility we observed. In a retrospective cohort of 105 patients with precancerous lesions, persistent infection was the only significant predictor of lesion progression (hazard ratio 4.39, 95% CI 2.20–8.78) [18], supporting confirmation of eradication during surveillance.

The contrast in reversibility between gastritis types—intestinal metaplasia was never lost in AIG (0%) but was no longer detected in 6.2% of *H. pylori* patients—is consistent with the description of AIG as an organ-specific disorder leading to progressive atrophy [7], although with five biopsies, lesions not detected at follow-up may partly reflect sampling variation. These findings support maintaining long-term surveillance in AIG regardless of symptomatic change. The near-absence of structural regression in our autoimmune cohort mirrors pediatric and adult follow-up series in which no endoscopic or histological improvement was documented and a substantial proportion of patients developed new metaplasia during surveillance [19,20,21], while rare partial regressions after *H. pylori* eradication in patients with coexistent infection [22] do not change the overall recommendation for indefinite monitoring.

### 4.4. PPI Effects and Clinical Risk Factors

No differential PPI effect between gastritis types was found (interaction *p* = 0.291), and PPI use was not associated with progression after adjustment (OR 0.86, 95% CI 0.34–2.17). Some population-based studies have reported associations between long-term PPI use and gastric cancer after *H. pylori* eradication [23], and in a large international AIG cohort prior PPI use was associated with gastric adenocarcinoma and type 1 neuroendocrine tumors [7]; our study used a histological endpoint and was not powered to exclude a modest effect, and PPI exposure was recorded only at the index endoscopy, without dose, so changes in PPI use during follow-up could not be taken into account.

The exploratory clinical risk score did not predict progression (AUC = 0.537). This finding applies to this internally derived, non-validated score, evaluated in the same cohort with only 15 patients in the highest category, and does not show that clinical risk factors in general lack predictive value. It is nevertheless consistent with the primacy of endoscopic and histological assessment over clinical stratification in managing gastric precancerous conditions [24].

### 4.5. Symptom–Histology Dissociation

The absence of correlation between symptom changes and histological progression (all |ρ| < 0.12, all *p* > 0.34) indicates that endoscopic monitoring cannot be replaced by clinical assessment, consistent with previous observations that symptoms are unreliable indicators of disease activity and cancer risk in gastritis [11,21].

### 4.6. Implications for Risk Stratification

Our findings suggest that gastritis etiology may be relevant to risk stratification. However, our endpoint was histological, gastric neoplasia was not assessed, and surveillance schedules were not compared, so these data cannot establish whether a specific surveillance interval is adequate for AIG. Current recommendations increasingly advocate risk-stratified rather than uniform intervals anchored on OLGA/OLGIM staging, as in the 2025 MAPS III update [6] and the 2025 Asian Pacific Association of Gastroenterology task force recommendations [25].

Risk-stratification frameworks could consider: (1) gastritis etiology, with recognition of AIG as a potentially higher-risk condition [8], (2) regional gastric cancer incidence as a population-level risk modifier, (3) *H. pylori* eradication status, and (4) baseline lesion extent. The exploratory clinical score and symptom changes evaluated here did not contribute to risk stratification. Emerging post-eradication markers such as map-like redness, observed in roughly one-quarter of patients at one year and associated with severe intestinal metaplasia [26], may further refine individualized surveillance once externally validated.

### 4.7. Study Strengths and Limitations

This study provides several methodological strengths. The systematic five-site biopsy protocol following Sydney System recommendations enabled topographic assessment essential for distinguishing corpus-predominant autoimmune patterns from antral-predominant *H. pylori* patterns. Single-pathologist evaluation throughout the study period ensured consistent histological interpretation, addressing inter-observer variability concerns. Median follow-up of 60 months (autoimmune) and 49 months (*H. pylori*) provided medium-term progression data, while the head-to-head comparison design directly addressed the evidence gap regarding differential risk between gastritis etiologies acknowledged by current guidelines.

Important limitations warrant acknowledgment. Because AIG was not suspected before the histopathology report, anti-parietal cell and anti-intrinsic factor antibodies were not available at the index endoscopy; a proportion of patients underwent confirmatory testing after the first biopsy, performed outside our hospital as part of routine care, but these results were not available for this study. In addition, these assays were not recommended at the time some patients were examined and were not supported by our hospital, and gastrin, chromogranin A, and vitamin B12 were measured inconsistently in routine care, so a sensitivity analysis restricted to serologically confirmed cases was not possible. Our classification relied on well-established corpus-predominant histological patterns that demonstrate high specificity for AIG when strict topographic criteria are applied, reflecting real-world diagnostic practice in resource-constrained settings. While this pragmatic approach aligns with diagnostic frameworks emphasizing histological patterns as primary criteria [11] and is supported by contemporary pathological descriptions that recognize corpus-restricted oxyntic atrophy with enterochromaffin-like (ECL) cell hyperplasia and antral sparing as the dominant diagnostic features when serology is unavailable [27], it introduces the possibility of including some patients with idiopathic corpus atrophy. This definition corresponds to that used in the largest international multicenter AIG cohort, which required atrophy of the oxyntic mucosa with antral sparing and no concurrent *H. pylori* infection and did not require antibody positivity [7]. Immunohistochemistry for ECL cell hyperplasia is available for part of the AIG cohort and will be analyzed separately. *H. pylori* serological testing to exclude past infection in the autoimmune group was not performed; however, the corpus-predominant pattern with antral sparing is atypical for *H. pylori*-associated atrophy, which characteristically shows antral-to-corpus progression, and spontaneous *H. pylori* clearance in adults is uncommon. Conversely, previous infection in the 27 baseline-negative comparator patients was inferred from the topographic pattern.

The retrospective single-center design limits generalizability. Patients undergoing repeat endoscopy may represent a higher-risk subset than the broader population with gastric precancerous lesions, potentially overestimating progression rates. Lesion severity was not graded, which precluded OLGA/OLGIM staging and assessment of worsening of pre-existing lesions. With five biopsies, patchy atrophy and intestinal metaplasia are subject to sampling variation, so lesions not detected at follow-up may not represent true regression. Histology was read in routine care by a single pathologist who was not blinded. Narrow-band imaging was not used in all examinations, although the biopsy protocol was identical throughout. Progression was ascertained at a single follow-up endoscopy whose timing was clinically determined, so the time of onset is unknown. Eradication was assessed at follow-up rather than by a timed test of cure. Clinical exposures, including PPI use, were recorded only at the index endoscopy. The symptom and risk scores were investigator-defined and not validated. Finally, the endpoint was histological; gastric neoplasia was not assessed. These findings require validation in prospective multicenter studies across diverse populations and healthcare systems.

## 5. Conclusions

This retrospective single-center study provides a head-to-head comparison of autoimmune and *H. pylori*-associated gastritis in an Eastern European cohort of patients who underwent repeat endoscopy, a selected population that may not represent all patients with chronic gastritis. In this cohort, histologically defined AIG was associated with more frequent histological progression, particularly development of glandular atrophy, and minimal reversibility compared with *H. pylori*-associated gastritis; these observational data do not establish the natural course of either condition. Progression in *H. pylori*-associated gastritis differed by observed infection status, but the design does not allow conclusions about the effect of eradication, whereas PPI use, an exploratory clinical risk score, and symptom changes were not associated with histological progression. These findings support considering gastritis etiology and baseline lesion extent in risk stratification but do not determine optimal surveillance intervals, which require prospective multicenter studies with serologically confirmed AIG, graded staging, and neoplastic endpoints.

## Figures and Tables

**Table 1:** Baseline Characteristics of Study Population.

Characteristic	AIG (*n* = 52)	*H. pylori* Gastritis (*n* = 64)	*p*-Value
**Demographics**			
Age, years (mean ± SD)	62.2 ± 11.3	59.5 ± 13.2	0.252
Female sex, *n* (%)	36 (69.2)	43 (67.2)	0.814
Follow-up, months, median (IQR)	60.0 (21.0–74.0)	49.0 (34.8–72.0)	0.874
**Clinical Risk Factors**			
PPI use, *n* (%)	26 (50.0)	16 (25.0)	0.005
Current smoking, *n* (%)	8 (15.4)	21 (32.8)	0.031
Diabetes mellitus, *n* (%)	11 (21.2)	8 (12.5)	0.210
NSAID use, *n* (%)	18 (34.6)	7 (10.9)	0.002
Anemia at baseline, *n* (%)	25 (48.1)	10 (15.6)	<0.001
**Baseline Histology**			
Inflammation grade, median (IQR) ᵃ	1.0 (1.0–1.0)	2.0 (1.0–2.0)	<0.001
Atrophy present, *n* (%)	9 (17.3)	10 (15.6)	0.808
Intestinal metaplasia, *n* (%)	8 (15.4)	7 (10.9)	0.478
Risk score, median (IQR) ᵇ	3.0 (2.0–3.2)	2.0 (1.0–2.0)	<0.001

ᵃ Graded 0–3 according to updated Sydney System. ᵇ Exploratory, investigator-defined composite score (0–7) including age > 50, PPI use, smoking, diabetes, thyroiditis, anemia, NSAID use. Categorical variables compared with the chi-square test without continuity correction, or Fisher’s exact test when any expected count was <5.

**Table 2:** Primary and lesion-specific outcomes and sensitivity analyses.

Outcome	AIG, *n*/*N* (%)	*H. pylori*, *n*/*N* (%)	Risk Difference, pp (95% CI)	Adjusted OR (95% CI); *p*
Primary: new atrophy or IM (all patients)	32/52 (61.5%)	22/64 (34.4%)	27.2 (8.9 to 43.1)	3.81 (1.53–9.49); *p* = 0.004
Incident atrophy (atrophy-free at baseline)	28/43 (65.1%)	17/54 (31.5%)	33.6 (13.7 to 50.1)	3.63 (1.47–8.98); *p* = 0.005
Incident IM (IM-free at baseline)	21/44 (47.7%)	15/57 (26.3%)	21.4 (2.6 to 38.7)	2.46 (1.01–6.01); *p* = 0.048
Sensitivity: comparator restricted to baseline *H. pylori*-positive (*n* = 37)				
New atrophy or IM	32/52 (61.5%)	17/37 (45.9%)	15.6 (−5.1 to 34.7)	2.36 (0.83–6.66); *p* = 0.106
Incident atrophy	28/43 (65.1%)	12/33 (36.4%)	28.8 (6.1 to 47.6)	3.02 (1.08–8.46); *p* = 0.036
Incident IM	21/44 (47.7%)	12/35 (34.3%)	13.4 (−8.2 to 33.1)	1.75 (0.64–4.75); *p* = 0.274
Sensitivity: PPI non-users only (*n* = 74)	15/26 (57.7%)	18/48 (37.5%)	20.2 (−3.3 to 41.0)	1.82 (0.64–5.16); *p* = 0.262

IM, intestinal metaplasia; pp, percentage points; OR, odds ratio (AIG vs. *H. pylori*-associated gastritis) from logistic regression adjusted for age, sex, follow-up duration, PPI use and smoking, plus baseline atrophy/IM for the all-patient outcome; the PPI non-user model omits PPI use.

**Table 3:** Logistic regression for new atrophy or intestinal metaplasia (*n* = 116).

Variable	Univariable OR (95% CI)	*p*	Multivariable OR (95% CI)	*p*
AIG (vs. *H. pylori*)	3.05 (1.43–6.53)	0.004	3.81 (1.53–9.49)	0.004
Age (per year)	1.02 (0.99–1.05)	0.322	1.01 (0.98–1.05)	0.451
Male sex	1.33 (0.61–2.90)	0.479	1.98 (0.75–5.23)	0.171
Follow-up (per year)	0.90 (0.74–1.08)	0.256	0.85 (0.67–1.08)	0.178
Atrophy/IM at baseline	0.08 (0.02–0.37)	0.001	0.06 (0.01–0.29)	<0.001
PPI use	1.24 (0.58–2.65)	0.575	0.86 (0.34–2.17)	0.750
Current smoking	0.51 (0.21–1.23)	0.136	0.44 (0.14–1.33)	0.144

Multivariable model includes all listed variables. Baseline atrophy/IM is inversely associated because patients with a pre-existing lesion cannot newly develop it.

**Table 4:** Transitions of precancerous lesions from baseline to follow-up. *p*-values for between-group comparisons using chi-square test without continuity correction, or Fisher’s exact test when any expected count was <5. *H. pylori*-associated group includes 37 patients with active infection at baseline who received eradication therapy, plus 27 patients with *H. pylori*-negative antral/pangastric patterns consistent with previous infection. McNemar’s test assesses within-group changes from baseline to follow-up. Net change = (prevalence at follow-up) − (prevalence at baseline). Lesions “not detected at follow-up” may reflect sampling variation rather than true regression.

Lesion Type	AIG (*n* = 52)	*H. pylori*-Associated Gastritis (*n* = 64)	*p*-Value
**GLANDULAR ATROPHY**			
Present at baseline, *n* (%)	9 (17.3%)	10 (15.6%)	0.808
Present at follow-up, *n* (%)	34 (65.4%)	20 (31.3%)	<0.001
Transitions:			
New development	28 (53.8%; 95% CI 40.5–66.7)	17 (26.6%; 95% CI 17.3–38.5)	0.003
Not detected at follow-up	3 (5.8%)	7 (10.9%)	0.508
Persistent	6 (11.5%)	3 (4.7%)	0.295
Never developed	15 (28.8%)	37 (57.8%)	0.002
Net change	+48.1 percentage points	+15.6 percentage points	-
McNemar’s test	*p* < 0.001	*p* = 0.066	-
**INTESTINAL METAPLASIA**			
Present at baseline, *n* (%)	8 (15.4%)	7 (10.9%)	0.478
Present at follow-up, *n* (%)	29 (55.8%)	18 (28.1%)	0.003
Transitions:			
New development	21 (40.4%; 95% CI 28.2–53.9)	15 (23.4%; 95% CI 14.7–35.1)	0.050
Not detected at follow-up	0 (0.0%)	4 (6.2%)	0.127
Persistent	8 (15.4%)	3 (4.7%)	0.062
Never developed	23 (44.2%)	42 (65.6%)	0.021
Net change	+40.4 percentage points	+17.2 percentage points	-
McNemar’s test	*p* < 0.001	*p* = 0.022	-

**Table 5:** Progression in *H. pylori*-associated gastritis by infection course.

*H. pylori* Subgroup	*n*	Median Follow-Up, Months (IQR)	Atrophy/IM at Baseline, *n*	New Atrophy or IM, *n*/*N* (%)	95% CI, %
Eradicated (positive → negative)	28	48 (38–63)	3	12/28 (42.9%)	26.5–60.9
Persistent infection (positive → positive)	9	52 (32–72)	1	5/9 (55.6%)	26.7–81.1
Baseline-negative, negative at follow-up	26	49 (35–74)	7	4/26 (15.4%)	6.2–33.5
Baseline-negative, positive at follow-up	1	72 (72–72)	0	1/1 (100.0%)	20.7–100.0

Infection status at follow-up was assessed histologically at the follow-up endoscopy; post-treatment confirmation was not systematic. Eradicated vs. persistent infection: *p* = 0.70 (Fisher’s exact test). CI, Wilson 95% confidence interval.

## Data Availability

The data presented in this study are available on request from the corresponding author due to ethical restrictions.
